# *XPG* gene rs751402 C>T polymorphism and cancer risk: Evidence from 22 publications

**DOI:** 10.18632/oncotarget.19421

**Published:** 2017-07-18

**Authors:** Haixia Zhou, Ting-Yan Shi, Wenwen Zhang, Qiwen Li, Jinhong Zhu, Jing He, Jichen Ruan

**Affiliations:** ^1^ Department of Hematology, The Second Affiliated Hospital and Yuying Children's Hospital of Wenzhou Medical University, Wenzhou 325027, Zhejiang, China; ^2^ Department of Obstetrics and Gynecology, Zhongshan Hospital, Fudan University, Shanghai 200032, China; ^3^ State Key Laboratory of Oncology in South China, Department of Radiation Oncology, Sun Yat-sen University Cancer Center, Collaborative Innovation Center for Cancer Medicine, Guangzhou 510060, Guangdong, China; ^4^ Molecular Epidemiology Laboratory and Department of Laboratory Medicine, Harbin Medical University Cancer Hospital, Harbin 150040, Heilongjiang, China; ^5^ Department of Pediatric Surgery, Guangzhou Institute of Pediatrics, Guangzhou Women and Children’s Medical Center, Guangzhou Medical University, Guangzhou 510623, Guangdong, China

**Keywords:** DNA repair, XPG, polymorphism, cancer susceptibility, meta-analysis

## Abstract

The *Xeroderma pigmentosum group G* (*XPG*) gene promotes recognition and excision of damaged DNA during the DNA repair process. We conducted a comprehensive search of the MEDLINE, EMBASE, and Chinese Biomedical databases for publications evaluating the association *XPG* gene rs751402 C>T polymorphism and overall cancer risk. Pooled odds ratios (ORs) and 95% confidence intervals (CIs) were adopted to assess the strength of the association. A total of 22 publications encompassing 10538 cases and 10511 control subjects were included in the final meta-analysis. We found the polymorphism to be associated with increased cancer risk (TT vs. CC: OR = 1.18, 95% CI = 1.01–1.38, *P* = 0.040; CT vs. CC: OR = 1.12, 95% CI = 1.01–1.24, *P* = 0.040; and CT/TT vs. CC: OR = 1.12, 95% CI = 1.002–1.26, *P* = 0.045). Stratification by cancer type indicated that this polymorphism may increase the risk of gastric cancer and hepatocellular carcinoma, which was further confirmed by a false-positive report probability analysis. Genotype-based mRNA expression provides further evidence that this polymorphism is associated with altered *XPG* mRNA expression. This meta-analysis suggests *XPG* gene rs751402 C>T polymorphism correlates with overall cancer risk, especially for gastric cancer and hepatocellular carcinoma.

## INTRODUCTION

According to an estimation by GLOBOCAN, approximately 14.1 million new cancer cases, including 8.2 million deaths, occurred worldwide in 2012 [1]. Approximately 4,292,000 new cancer cases and 2,814,000 cancer deaths occurred in China in 2015, with lung, gastric, esophageal, and liver cancer being the most commonly diagnosed and the leading causes of death [2]. Risk factors for the leading causes of cancer-related deaths are tobacco consumption, overweight/obesity, physical inactivity, and infection [1]. Genetic factors should also be considered [3–8].

Human DNA repair genes maintain the integrity and stability of genomic DNA, consequently preventing carcinogenesis and influencing clinical outcomes [9, 10]. Many genes promote the diverse DNA repair pathways, including the nucleotide excision repair (NER) pathway [11]. The NER pathway consists of damage recognition, demarcation, dual incision, and gap filling and can repair a variety of damaged DNA [12]. The NER pathway is the main mechanism for the removal of DNA adducts and lesions caused by chemical adducts [13]. Polymorphisms of the genes from the NER pathway might activate cancer risk alteration [14]. As one of the eight core genes in the NER pathway, *Xeroderma pigmentosum* group G (*XPG*), which is also known as excision repair cross-complementing group 5 (ERCC5), can recognize and excise DNA lesions on the 3′ side to repair damaged DNA [15].

*XPG* gene polymorphisms were reported to be associated with the susceptibility of various types of cancers [16–18]. Thus, most of the investigations were focused on rs17655 G>C (Asp1104His). The association between *XPG* gene rs751402 C>T polymorphism (located at the 5′ untranslational region) and cancer risk has been investigated in several studies [19–40], but the findings were contradictory and inconclusive. Therefore, we performed this meta-analysis with all eligible publications to comprehensively evaluate the association of *XPG* gene rs751402 C>T polymorphism with overall cancer risk.

## RESULTS

### Characteristics of eligible publications

**Figure 1:**
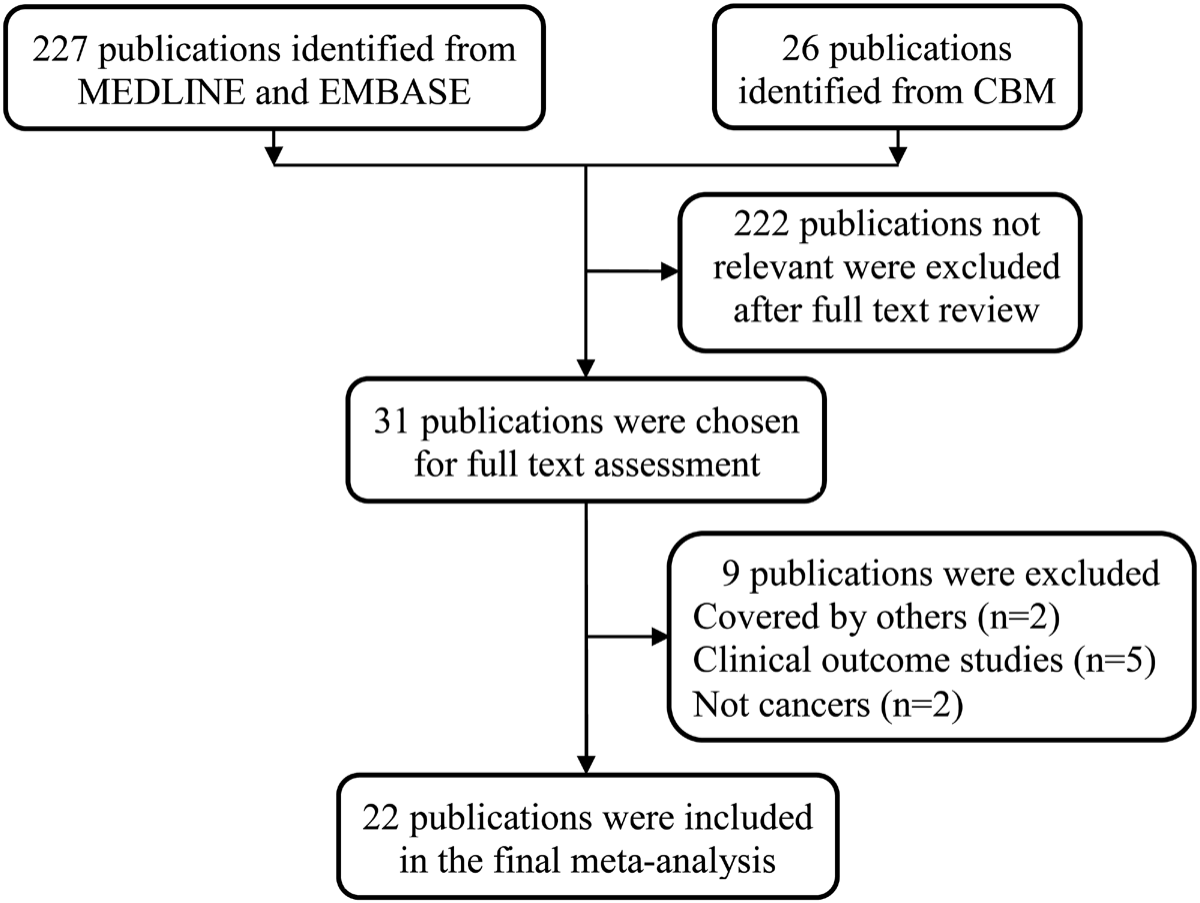
Flowchart of the included publications

As shown in Figure 1, 227 publications were identified from MEDLINE and EMBASE and 26 additional publications in Chinese were identified from the Chinese Biomedical (CBM) database. After reviewing the abstracts and the full texts, we excluded 222 publications and selected 31 publications with studies of the rs751402 C>T polymorphism for further full-text review. Among these publications, nine were excluded because two studies were repetitive, five studies were clinical outcome studies, and two studies were not on cancers. In the final meta-analysis, 22 publications with studies of 10588 cases and 10511 control subjects were identified, with the duplicated samples counted only once. The characteristics of the included publications are showed in Table 1. In these publications, sample sizes ranged from 96 to 1900 cases and from 101 to 1977 control subjects. Among the studies, 10 focused on gastric cancer [21, 23, 27, 29, 30, 32–34, 38, 39], three focused on breast cancer [25, 35, 36], two focused on hepatocellular carcinoma [20, 37], and one each focused on lung cancer [19], oral squamous cell carcinoma [22], salivary gland tumor [24], nasopharyngeal carcinoma [26], neuroblastoma [28], colorectal cancer [31], and prostate cancer [40]. Of the publications, 12 had quality scores higher than nine, and 10 had quality scores of no more than nine.

**Table 1:** Characteristics of the included studies in the final meta-analysis

Name	Year	Cancer type	Region	Ethnicity	Design	Genotype method	Case				Control				MAF	HWE	Score
							CC	CT	TT	All	CC	CT	TT	All			
Shao	2007	Lung	China	Asian	HB	Taqman	433	429	105	967	448	425	110	983	0.33	0.544	11
Yoon	2011	HCC	Taiwan	Asian	HB	Taqman	33	52	11	96	167	137	32	336	0.30	0.614	6
Duan	2012	Gastric	China	Asian	HB	MassARRAY	172	181	47	400	206	165	29	400	0.28	0.605	11
Zavras	2012	OSCC	Taiwan	Asian	HB	Taqman	98	110	31	239	167	137	32	336	0.30	0.614	9
He	2013	Gastric	China	Asian	HB	Taqman	486	491	148	1125	560	499	137	1196	0.32	0.110	13
Meng	2013	Salivary gland	China	Asian	HB	PCR-RFLP	59	63	11	133	64	55	23	142	0.36	0.065	8
Na	2015	Breast	China	Asian	HB	PCR-RFLP	128	152	45	325	137	147	41	325	0.35	0.872	10
Sun	2015	NPC	China	Asian	HB	PCR-LDR	17	118	237	372	19	117	235	371	0.79	0.377	11
Chen	2016	Gastric	China	Asian	HB	Taqman	286	313	93	692	351	331	89	771	0.33	0.416	11
He	2016	Neuroblastoma	China	Asian	HB	Taqman	96	114	38	248	208	241	82	531	0.38	0.380	10
Feng	2016	Gastric	China	Asian	HB	PCR-RFLP	70	83	24	177	101	107	28	236	0.35	0.967	6
Guo	2016	Gastric	China	Asian	HB	PCR-RFLP	47	73	22	142	117	136	21	274	0.32	0.029	5
Hua	2016	Colorectal	China	Asian	HB	Taqman	792	860	248	1900	724	952	301	1977	0.39	0.680	10
Hua	2016	Gastric	China	Asian	HB	Taqman	426	555	161	1142	433	551	189	1173	0.40	0.537	11
Li	2016	Gastric	China	Asian	HB	PCR-RFLP	88	106	22	216	95	103	18	216	0.32	0.174	8
Lu	2016	Gastric	China	Asian	HB	PCR-RFLP	69	91	24	184	87	97	22	206	0.34	0.510	6
Ma	2016	Breast	China	Asian	HB	PCR-RFLP	127	150	43	320	107	101	28	236	0.33	0.580	7
Wang	2016	Breast	China	Asian	HB	PCR-RFLP	90	10	1	101	51	39	11	101	0.30	0.398	9
Wang	2016	HCC	China	Asian	PB	MassARRAY	70	81	18	169	232	185	60	477	0.32	0.018	12
Yang	2016	Gastric	China	Asian	HB	PCR-RFLP	49	73	33	155	103	111	32	246	0.36	0.807	6
Zhou	2016	Gastric	China	Asian	HB	PCR-LDR	174	196	61	431	193	193	46	432	0.33	0.827	12
Wang	2017	Prostate	China	Asian	HB	Taqman	442	458	104	1004	477	467	111	1055	0.33	0.834	10

MAF, minor allele frequency; HWE, Hardy-Weinberg equilibrium; HCC, hepatocellular carcinoma; OSCC, oral squamous cell carcinoma; NPC, nasopharyngeal carcinoma; HB, hospital based; PB, population based; PCR-RFLP, polymerase chain reaction-restriction fragment length polymorphism; PCR-LDR, polymerase chain reaction- ligase detection reaction.

### Meta-analysis results

**Figure 2:**
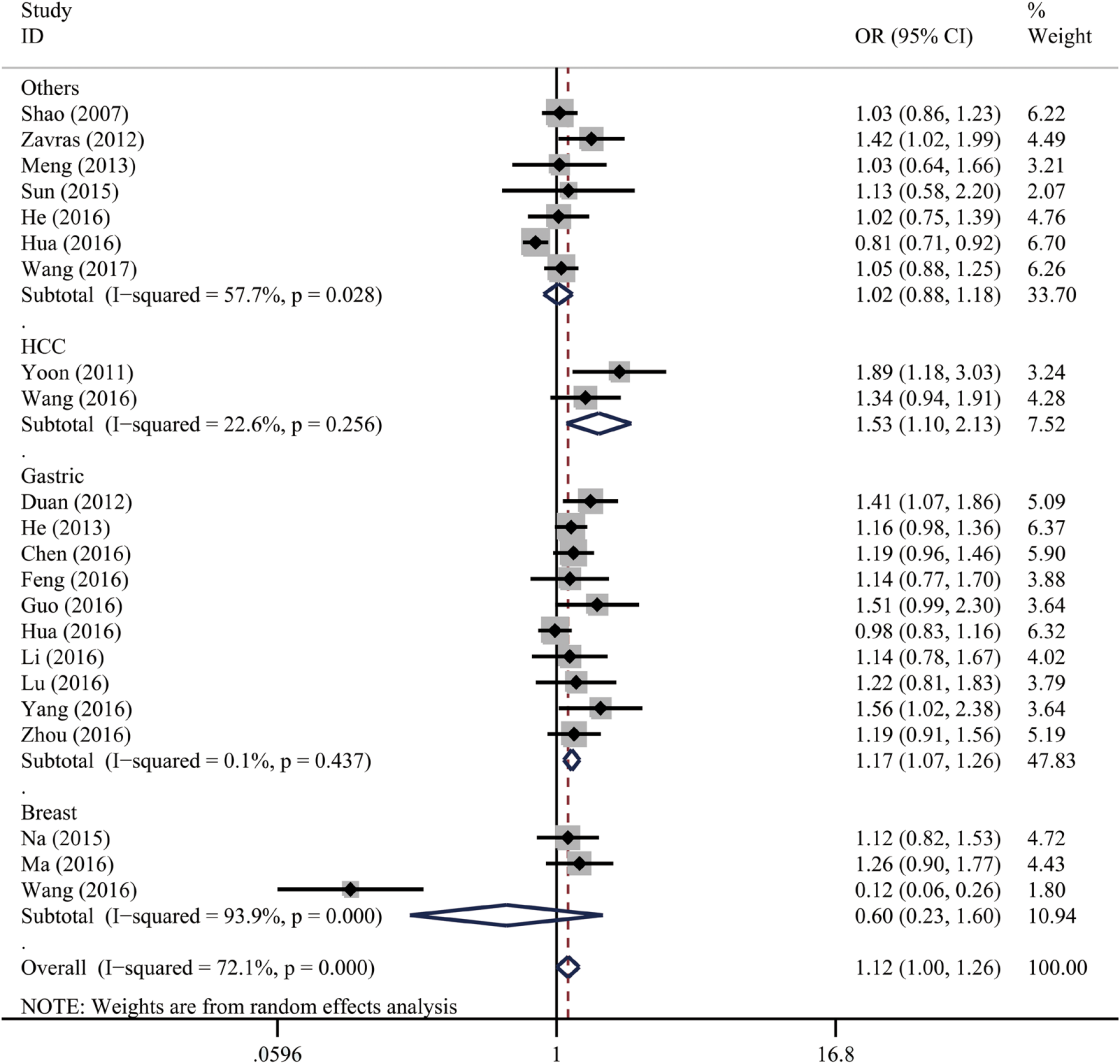
Stratification analysis for the association between *XPG* gene rs751402 C>T polymorphism and overall cancer risk by cancer type under the dominant model (CT/TT vs. CC) For each publication, the estimation of OR and its 95% CI are plotted with a box and a horizontal line. The diamonds represent the pooled ORs and 95% CIs.

As shown in Table 2, significant heterogeneity was presented in all genetic models. As a result, we adopted a random-effect model for all the analyses. We found the *XPG* gene rs751402 C>T polymorphism associated with increased overall cancer risk (TT vs. CC: odds ratio [OR] = 1.18, 95% confidence interval [CI] =1.01–1.38; CT vs. CC: OR = 1.12, 95% CI = 1.01–1.24; and CT/TT vs. CC: OR = 1.12, 95% CI = 1.002–1.26). As shown in Figure 2, stratification analysis indicated that this polymorphism was associated with increased risk of gastric cancer (TT vs. CC: OR = 1.38, 95% CI = 1.12-1.70; CT vs. CC: OR = 1.14, 95% CI = 1.05–1.24; TT vs. CC/CT: OR = 1.27, 95% CI = 1.06-1.51; CT/TT vs. CC: OR = 1.17, 95% CI = 1.08–1.26; and T vs. C: OR = 1.17, 95% CI = 1.07–1.27) and hepatocellular carcinoma (CT vs. CC: OR = 1.61, 95% CI = 1.19–2.17; and CT/TT vs. CC: OR=1.53, 95% CI=1.10-2.13). The stratification analysis did not reveal a significant difference between the two strata in any genetic model by quality score.

**Table 2:** Meta-analysis of the association between *XPG* gene rs751402 C>T polymorphism and overall cancer risk

Variables	No. of studies	Sample size	Homozygous		Heterozygous		Recessive		Dominant		Allele	
			TT vs. CC		CT vs. CC		TT vs. CT+CC		CT+TT vs. CC		T vs. C	
			OR (95% CI)	*P*^het^	OR (95% CI)	*P*^het^	OR (95% CI)	*P*^het^	OR (95% CI)	*P*^het^	OR (95% CI)	*P*^het^
All	22	10538/10511	**1.18 (1.01–1.38)**	<0.001	**1.12 (1.01–1.24)**	<0.001	1.09 (0.97–1.23)	0.009	**1.12 (1.002–1.26)**	<0.001	1.09 (1.00–1.18)	<0.001
Cancer type												
Gastric	10	4664/5150	**1.38 (1.12–1.70)**	0.020	**1.14 (1.05–1.24)**	0.936	**1.27 (1.06–1.51)**	0.053	**1.17 (1.08–1.26)**	0.437	**1.17 (1.07–1.27)**	0.043
Breast	3	746/662	0.79 (0.31–1.98)	0.010	0.64 (0.26–1.58)	<0.001	0.87 (0.43–1.79)	0.044	0.60 (0.23–1.60)	<0.001	0.63 (0.29–1.35)	<0.001
HCC	2	265/813	1.24 (0.73–2.12)	0.262	**1.61 (1.19–2.17)**	0.373	0.96 (0.62–1.49)	0.398	**1.53 (1.10–2.13)**	0.256	1.26 (0.97–1.63)	0.220
Others	7	4863/5395	0.95 (0.78–1.16)	0.082	1.03 (0.89–1.18)	0.071	0.94 (0.82–1.07)	0.270	1.02 (0.88–1.18)	0.028	0.99 (0.90–1.10)	0.025
Quality score												
>9	12	8775/9691	1.08 (0.93–1.25)	0.011	1.06 (0.98–1.17)	0.063	1.02 (0.92–1.14)	0.137	1.08 (0.98–1.19)	0.007	1.05 (0.98–1.14)	0.002
≤9	10	1763/2329	1.34 (0.95–1.89)	0.009	1.13 (0.86–1.48)	<0.001	1.21 (0.90–1.62)	0.029	1.12 (0.84–1.51)	<0.001	1.07 (0.85–1.35)	<0.001

OR, odds ratio; CI, confidence interval; Het, heterogeneity; HCC, hepatocellular carcinoma.

### False-positive report probability analysis for significant findings

We performed false-positive report probability (FPRP) analysis for all significant findings and confirmed that the findings were significant at the priority of 0.1 for gastric cancer and hepatocellular carcinoma (Table 3).

**Table 3:** False-positive report probability analysis values for the noteworthy findings

Genotype	Crude OR (95% CI)	*P*^a^	Statistical power^b^	Prior probability				
				0.25	0.1	0.01	0.001	0.0001
Overall cancer risk								
TT vs. CC	1.18 (1.01–1.38)	0.040	1.000	**0.107**	0.264	0.798	0.976	0.998
CT vs. CC	1.12 (1.01–1.24)	0.040	1.000	**0.106**	0.263	0.797	0.975	0.997
CT/TT vs. CC	1.12 (1.002–1.26)	0.047	1.000	**0.123**	0.296	0.822	0.979	0.998
Hepatocellular carcinoma								
CT vs. CC	1.61 (1.19–2.17)	0.002	0.394	**0.013**	**0.038**	0.305	0.816	0.978
CT/TT vs. CC	1.53 (1.10–2.13)	0.011	0.608	**0.050**	**0.137**	0.636	0.946	0.994
Gastric cancer								
TT vs. CC	1.38 (1.12–1.70)	0.002	1.000	**0.007**	**0.019**	**0.179**	0.687	0.956
CT vs. CC	1.14 (1.05–1.24)	0.003	1.000	**0.008**	**0.024**	0.213	0.732	0.965
TT vs. CT/CC	1.27 (1.06–1.51)	0.010	1.000	**0.030**	**0.085**	0.506	0.912	0.990
CT/TT vs. CC	1.17 (1.08–1.26)	<0.001	1.000	**0.001**	**0.002**	**0.019**	**0.161**	0.658
T vs. C	1.17 (1.07–1.27)	0.001	1.000	**0.002**	**0.006**	**0.063**	0.404	0.871

OR, odds ratio; CI, confidence interval.

^a^A *χ^2^* test was used to evaluate the distributions of genotype frequency.

^b^Statistical power was calculated by use of the number of observations in the subgroup and *P* values in this table.

### The genotype-based mRNA expression for *XPG* gene rs751402 C>T polymorphism

As shown in Table 4, the rs751402T allele carriers were associated with decreased *XPG* mRNA expression among Asians (not significant), Africans (TT vs. CC: *P* = 0.029), and Caucasians (TT vs. CC: *P* = 0.013; and TT vs. CC/CT: *P* = 0.011), as well as all subjects (TT vs. CC: *P* = 0.010; and TT vs. CC/CT: *P* = 0.008).

**Table 4:** *XPG* gene mRNA expression by the genotypes of rs751402 C>T^a^

Population	Genotypes	No.	Mean ± SD	*P*^b^	*P*_trend_^c^
Asian	CC	30	9.79 ± 0.21		0.409
	CT	47	9.76 ± 0.22	0.537	
	TT	13	9.69 ± 0.23	0.188	
	Dominant	60	9.75 ± 0.22	0.352	
	Recessive	77	9.77 ± 0.22	0.233	
CEU	CC	54	9.72±0.23		**0.039**
	CT	29	9.70±0.22	0.823	
	TT	7	9.48±0.15	**0.013**	
	Dominant	36	9.66±0.22	0.271	
	Recessive	83	9.71±0.23	**0.011**	
YRI	CC	35	9.86±0.16		0.100
	CT	43	9.82±0.17	0.245	
	TT	12	9.75±0.14	**0.029**	
	Dominant	55	9.80±0.17	0.094	
	Recessive	78	9.84±0.17	0.074	
All	CC	119	9.78 ± 0.22		**0.030**
	CT	119	9.77 ± 0.21	0.693	
	TT	32	9.67 ± 0.21	**0.010**	
	Dominant	151	9.75 ± 0.21	0.220	
	Recessive	238	9.77 ± 0.21	**0.008**	

^a^The rs751402 C>T genotypes data were obtained from the HapMap Phase II Release 23 data, and *XPG* mRNA expression levels were from EBV-transformed lymphoblastoid cell lines from 270 individuals.

^b^Two-side Student’s *t-*test within the stratum.

^c^*P* values for the trend test of the *XPG* gene mRNA expression among three genotypes for rs751402 C>T from a general linear model.

### Sensitivity analysis and publication bias

**Figure 3:**
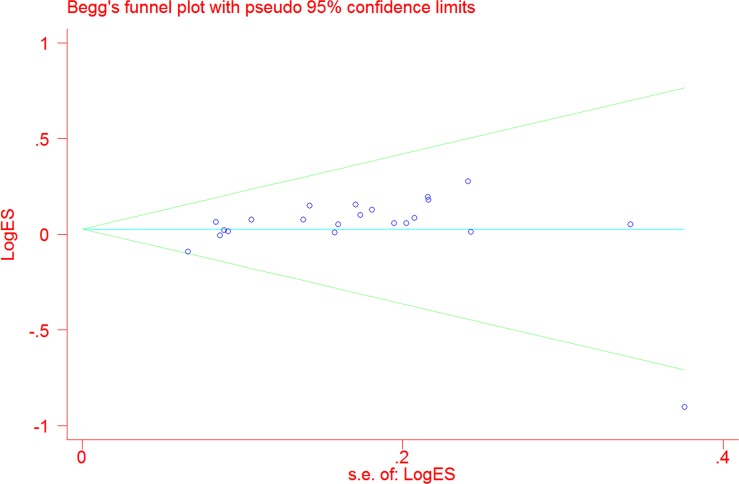
Funnel plot for the association between *XPG* gene rs751402 C>Tpolymorphism and overall cancer risk under the dominant model (CT/TT vs. CC)

By omitting each publication once in every genetic model in the sensitivity analysis, we did not find any individual publication that could significantly alter the pooled ORs, which indicated that our data were stable and trustworthy. As shown in Figure 3, no obvious publication bias was observed for rs751402 C>T polymorphism (TT vs. CC: *P* = 0.111; CT vs. CC: *P* = 0.251; TT vs. CT/CC: *P* = 0.236; CT/TT vs. CC: *P* = 0.249; and T vs. C: *P* = 0.298).

### Trial sequential analysis

**Figure 4:**
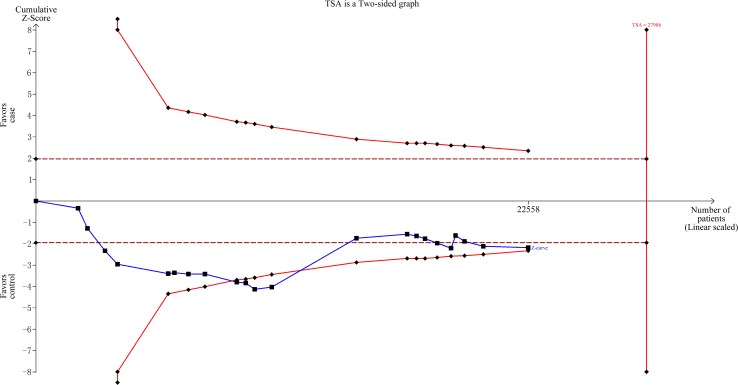
Trial sequential analysis for *XPG* gene rs751402 C>T polymorphism under the dominant model

As shown in Figure 4, we observed that the cumulative z-curve crossed the monitoring boundary before reaching the required sample size, indicating the sample size was sufficient and no further investigation was needed to verify the results.

## DISCUSSION

In the current meta-analysis, we investigated all available publications that contained studies of the association between *XPG* gene rs751402 C>T polymorphism and cancer risk. The pooled results suggest that this polymorphism is associated with increased cancer risk, especially for gastric cancer and hepatocellular carcinoma.

The *XPG* gene, which is located at 13q33 and consists of 15 exons, promotes the removal of damaged DNA in the NER process [41]. When DNA repair capability is decreased, cells might fail to repair the damage. As DNA mutations accumulate, carcinoma might occur [9, 21]. The *XPG* gene is an essential component of the NER pathway, and it activates the cleavage of DNA on the 3′ side of the lesion [42]. Studies reported that the *XPG* gene promotes cellular processes such as RNA polymerase II transcription and transcription-coupled DNA repair [43]. *XPG* gene polymorphisms might affect the expression or function of the XPG protein. Studies in several publications investigated the function of *XPG* gene rs751402 C>T polymorphism in cancer susceptibility. However, inconsistent results have been reported. Duan et al. [21] found that this polymorphism might increase the risk of gastric cancer in a study of 403 gastric cancer cases and 403 healthy control subjects. This association was also confirmed in gastric cancer by Yang et al. [38] in a study of 155 gastric cancer cases and 246 healthy control subjects, in hepatocellular carcinoma by Yoon et al. [20], and in oral squamous cell carcinoma by Zavras et al. [22]. Hua et al. [31] found that this polymorphism might be associated with decreased colorectal cancer susceptibility by studying 1901 colorectal cases and 1976 control subjects, and might have no effect in gastric cancer, as determined by 1142 cases and 1173 control subjects. Others found that this polymorphism might have weak effects on cancer susceptibility. The controversy can possibly be ascribed to the small sample size as well as cancer differences. To overcome the limitations of a single study and to reduce the likelihood of random errors being responsible for false-positive or false-negative associations, we performed the current meta-analysis to assess the association between *XPG* gene rs751402 C>T polymorphism and overall cancer susceptibility. We included 22 available publications, encompassing 10588 cases and 10511 control subjects, and found that this polymorphism was associated with increased overall cancer risk, especially for gastric cancer and hepatocellular carcinoma. We also performed FPRP analysis to confirm that the significant associations were trustworthy and robust. In addition, the genotype-based mRNA expression analysis as performed also indicated that this polymorphism might be associated with *XPG* gene mRNA expression alteration.

The current meta-analysis has five advantages. First, we searched the latest publications and we also included the publications written in Chinese. Second, we assessed the quality of each investigation and conducted stratification analysis by the quality score to search for publication bias. Third, we performed genotype-based mRNA expression analysis to provide further evidence that the rs751402 C>T polymorphism can influence the expression of the *XPG* gene. Fourth, we performed FPRP analysis, which can confirm whether the significant associations are trustworthy and robust. Fifth, we performed TSA to strengthen the robustness and minimize random errors of our conclusions.

Although in the present study we performed the latest and largest meta-analysis for assessing the association between *XPG* gene rs751402 C>T polymorphism and overall cancer susceptibility, four limitations must be considered. First, because of the heterogeneity in the current meta-analysis, the conclusions on the overall cancer risk should be interpreted cautiously. Second, the results of this study were based on the unadjusted ORs, which might suppress the final results. Third, all the study subjects were Asians. Other ethnicities are needed as subjects in future studies. Fourth, despite the adequacy of the total number of publications, the number of publications that contain studies for some cancers were inadequate. Investigations into other cancers are needed.

Our meta-analysis found that *XPG* gene rs751402 C>T polymorphism is associated with increased overall cancer risk, especially with respect to gastric cancer and hepatocellular carcinoma. Investigations of different cancers and ethnicities are needed to validate our findings.

## MATERIALS AND METHODS

### Publication search

We systematically searched publications from the MEDLINE, EMBASE, and CBM databases (the last search was updated April 28, 2017) using the following search terms: “cancer or carcinoma or tumor or neoplasm,” “excision repair cross-complementing group 5 or *ERCC5* or xeroderma pigmentosum group G or *XPG* or rs751402,” and “polymorphism or variant or single nucleotide polymorphism (SNP) or variation.” We also manually searched the reference lists of the articles in the included publications.

### Inclusion and exclusion criteria

The studies in the included publications met the following criteria: (1) the study evaluated the association between *XPG* gene rs751402 C>T polymorphism and cancer risk, (2) the study was on human beings, (3) the study was a case-control or cohort design, (4) sufficient data were provided to calculate the ORs and 95% CIs, and (5) the study was published in English or Chinese.

Exclusion criteria were (1) the study was not a case-control design, (2) the study was duplicated from previous studies, (3) articles were case reports or review articles, and (4) the studies were without detailed genotype data.

### Data extraction and quality assessment

Two authors (Haixia Zhou and Ting-Yan Shi) performed the publication search and data extraction independently. The extracted information includes surname of the first author, publication year, cancer type, country of origin, ethnicity, genotyping methods, and numbers of cases and control subjects with rs751402 CC, CT and TT genotypes. We assessed the quality of each publication based on the quality score assessment [44]. All contradictory information was discussed and resolved through consensus when necessary.

### Genotype-based mRNA expression analysis

To determine whether the *XPG* gene rs751402 C>T polymorphism can influence expression of the *XPG* gene, we conducted genotype-based mRNA expression analysis as previously described [3, 45, 46]. Genotype data of *XPG* gene rs751402 C>T polymorphism for 270 individuals were obtained from HapMap Phase II Release 23. The mRNA expression data for the corresponding individuals were from SNPexp [47].

### Statistical analysis

Pooled ORs and 95% CIs were used to investigate the strength of the association between *XPG* gene rs751402 C>T polymorphism and overall cancer risk under the homozygous (TT vs. CC), heterozygous (CT vs. CC), recessive (TT vs. CT+CC), dominant (CT+TT vs. CC), and allele contrast (T vs. C) models. A goodness-of-fit χ^2^ test was adopted to assess the Hardy-Weinberg equilibrium for the control subjects. Stratification analysis was carried out by cancer type (publications with no more than two were merged as the Others Group) and quality score (>9 and ≤9). Heterogeneities were assessed by χ^2^-based Q test, and a fixed-effect model was adopted when *P* > 0.1. Otherwise, the random-effect model was applied [48]. Sensitivity analysis was then conducted by omitting each publication in turn to evaluate the stability of the overall results. Potential publication bias was assessed by Begg’s funnel plot [49] and Egger’s linear regression test [50]. FPRP and TSA were as previously described [8]. All the statistics were two-sided, and *P* < 0.05 was statistically significant. All statistical analyses were performed by the STATA software (Version 11.0; Stata Corporation, College Station, TX).
